# Supplementary material for: Managing STEMIs without a Catheterization Lab: A Simulated Scenario to Improve Emergency Clinician Recognition and Execution of Thrombolysis in the Setting of Rural STEMI Management
Source: J Educ Teach Emerg Med. 2024 Apr 30;9(2):S55–77. doi: 10.21980/J8K933 (PMC11068321; doi:10.21980/J8K933)
Supplement: Supplementary file 1 [file jetem-9-2-S55-supp1.pptx]

## Slide 1
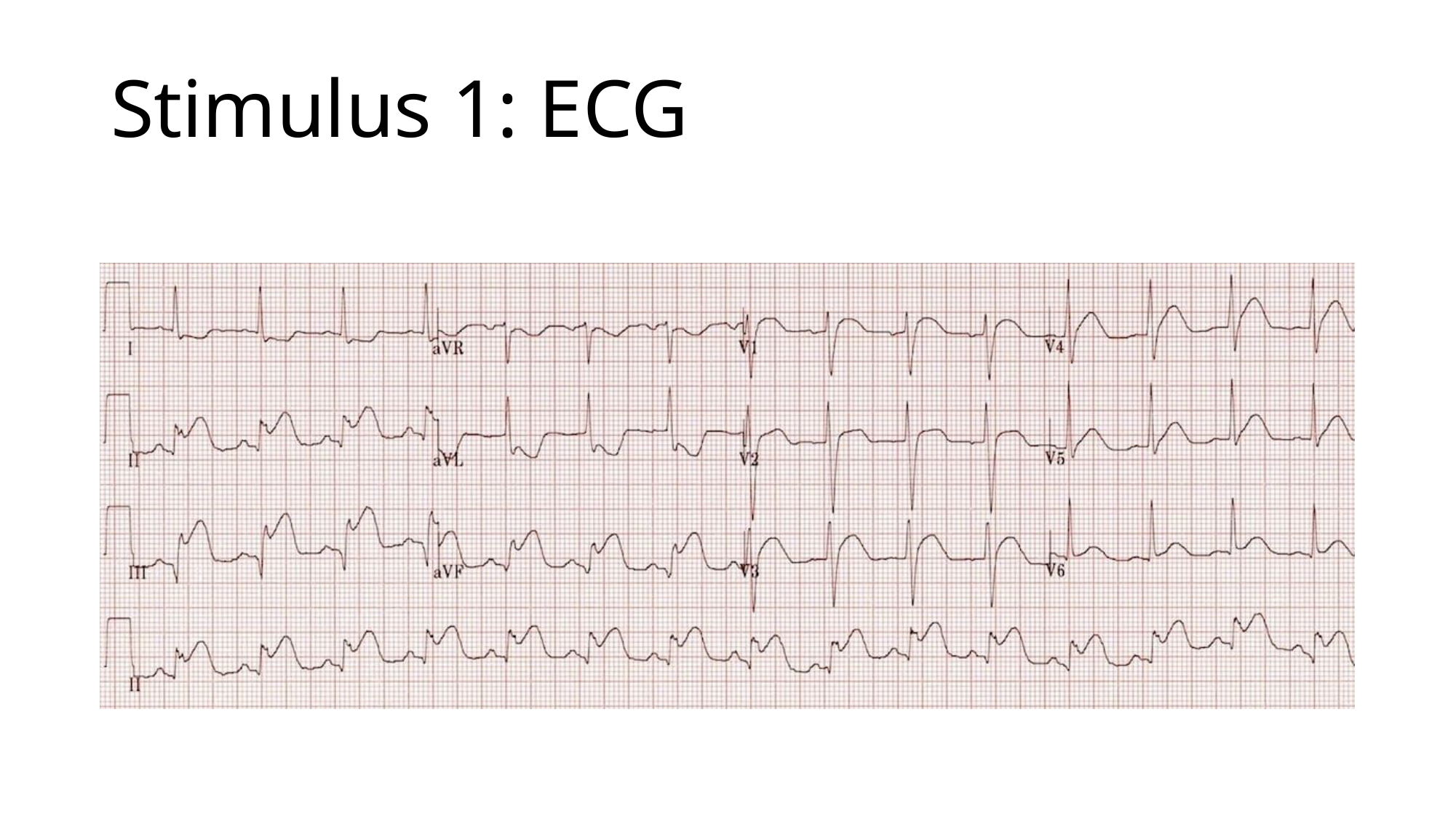

# Stimulus 1: ECG

## Slide 2
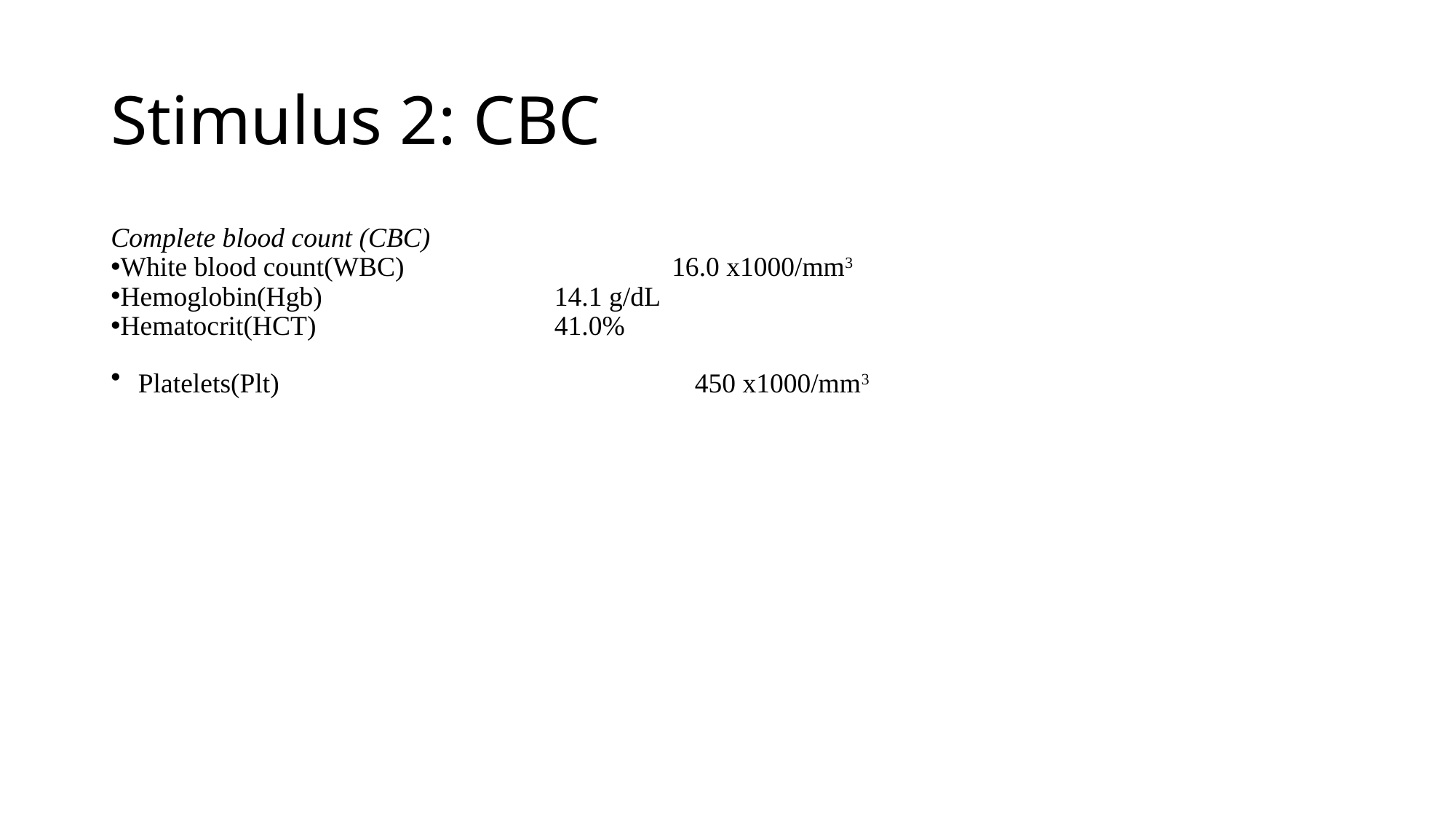

# Stimulus 2: CBC
Complete blood count (CBC)
White blood count(WBC)		 16.0 x1000/mm3
Hemoglobin(Hgb)			 14.1 g/dL
Hematocrit(HCT)			 41.0%
Platelets(Plt)	 450 x1000/mm3

## Slide 3
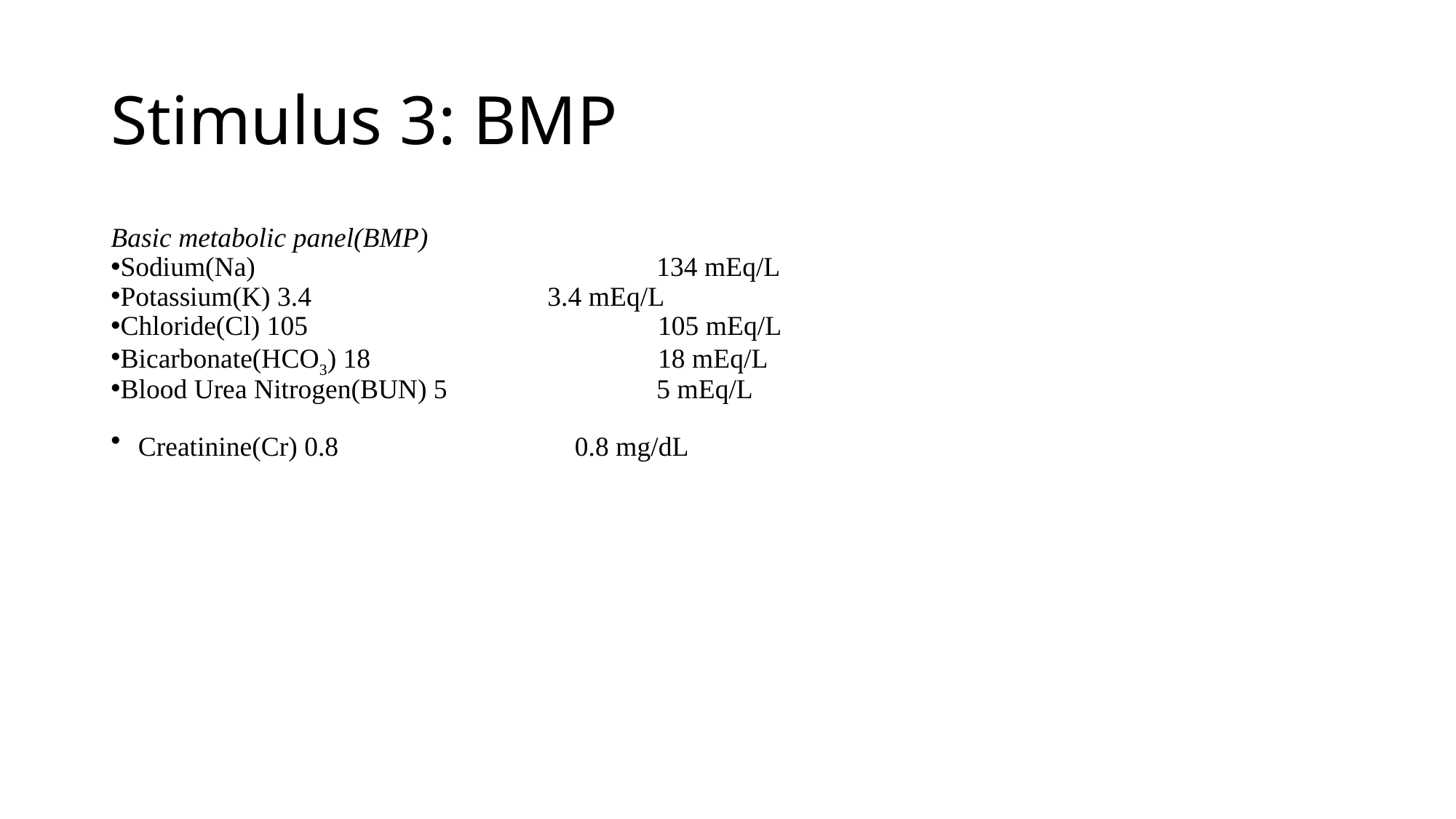

# Stimulus 3: BMP
Basic metabolic panel(BMP)
Sodium(Na) 				134 mEq/L
Potassium(K) 3.4			3.4 mEq/L
Chloride(Cl) 105			 105 mEq/L
Bicarbonate(HCO3) 18		 18 mEq/L
Blood Urea Nitrogen(BUN) 5		5 mEq/L
Creatinine(Cr) 0.8			0.8 mg/dL

## Slide 4
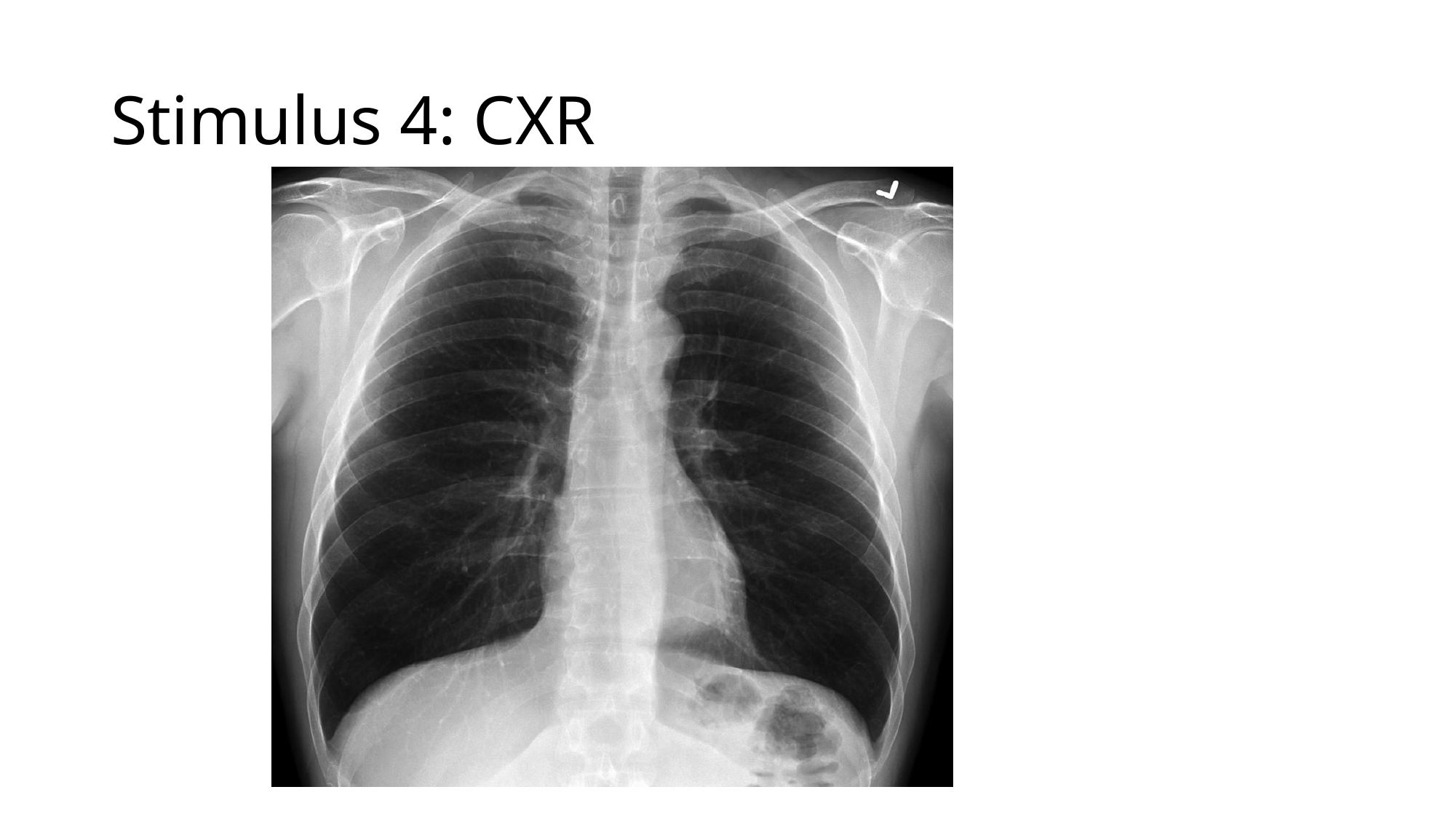

# Stimulus 4: CXR

## Slide 5
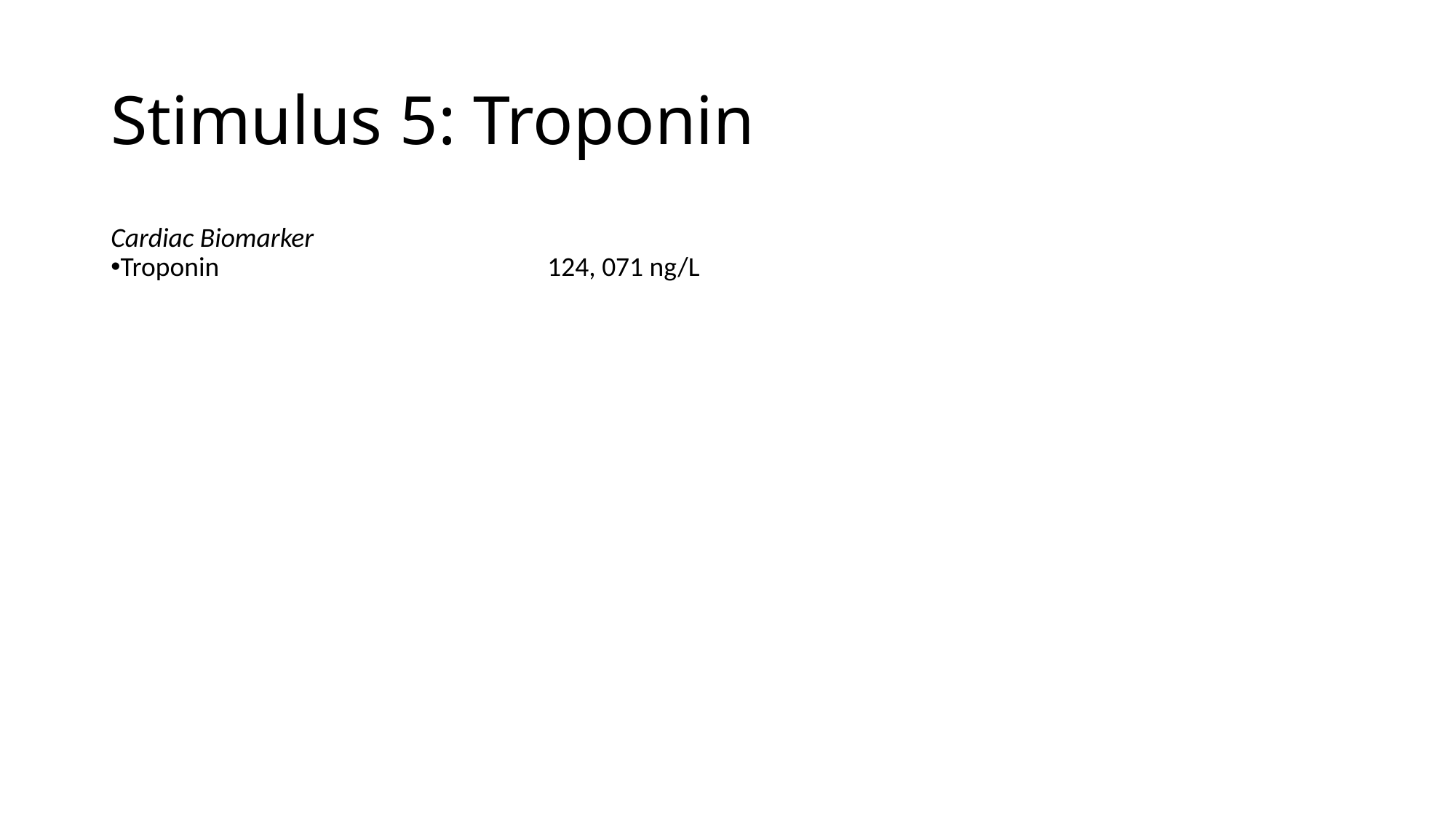

# Stimulus 5: Troponin
Cardiac Biomarker
Troponin				124, 071 ng/L
